# The Countdown to 2020: measuring progress in neglected tropical diseases

**DOI:** 10.1016/S2214-109X(15)00319-8

**Published:** 2016-02-04

**Authors:** Kebede Deribe

**Affiliations:** Brighton and Sussex Medical School, Brighton BN19PX, UK; and School of Public Health, Addis Ababa University, Addis Ababa, Ethiopia, kebededeka@yahoo.com

The London Declaration on Neglected Tropical Diseases^1^ marked its fourth year on Jan 30, 2016. The declaration represents a coordinate effort to control or eliminate ten of the neglected diseases by 2020, and has already led to important coordination and partnership, and mobilised considerable resources. Although the progress made so far is to be celebrated, now is the time to count down to 2020 and start monitoring the progress and trends towards achieving the control and elimination targets.

First, progress towards the 2020 targets should be measured through collation, presentation, and analysis of standardised core metrics that track country progress. Elimination and control targets in the WHO Roadmap could serve as initial standardised metrics.^2^ Efforts are being made to measure the progress and forecast future trends, but most efforts are disease-specific and occur at a global scale.^3^ Therefore, comprehensive measurements to track the goal of the declaration are needed. Country-specific case studies, considering different aspects of policy, programming, and intervention would provide the whole picture of the analysis needed for the countdown to 2020. Focus on assessment from a multidimensional aspect and pinpointing of bottlenecks will help to identify the gaps in implementation and point out areas that need improvement.

Second, development of standardised methods to measure the progress towards reaching the targets is crucial. Modelling methods, which use the available data to forecast future trends will be important for understanding how countries are progressing towards achieving the goals of the London declaration. These methods will enable comparison of the outputs of such an analysis, and several sources of data would be needed to provide continuous high-quality data for analysis. Collaboration with national surveys, such as the demographic and health surveys and several indicators surveys, would be crucial for achieving this goal.

Third, establishment of a global independent monitoring group would be essential to assess country-specific reports and set up an accountability framework.^4^ The group would have an important role in the definition of the core methods to monitor progress and develop a framework for country assessment mechanisms. This approach will help to further improve the quality of country-level reports.

As we go further down the road, understanding if we are on track to achieve the targets is necessary. Standardised metrics, continued monitoring, and comprehensive country-specific case studies will be crucial to advance the goal of the declaration.
